# Common MIR146A Polymorphisms in Chinese Ankylosing Spondylitis Subjects and Controls

**DOI:** 10.1371/journal.pone.0137770

**Published:** 2015-09-14

**Authors:** Zhenmin Niu, Jiucun Wang, Hejian Zou, Chengde Yang, Wei Huang, Li Jin

**Affiliations:** 1 State Key Laboratory of Genetic Engineering and Ministry of Education Key Laboratory of Contemporary Anthropology, Collaborative Innovation Center for Genetics and Development, School of Life Sciences, Fudan University, No. 2005 Songhu Road, Shanghai, 200438, China; 2 Shanghai-MOST Key Laboratory of Health and Disease Genomics, Chinese National Human Genome Center and Shanghai Industrial Technology Institute (SITI), No. 250 Bibo Road, Shanghai, 201203, China; 3 Division of Rheumatology, Huashan Hospital, Fudan University, No 12 Middle Wulumuqi Road, Shanghai, 200040, China; 4 Division of Rheumatology, Renji Hospital, Shanghai Jiaotong University, No 145 Middle Shandong Road, Shanghai, 200001, China; University of East London, UNITED KINGDOM

## Abstract

Common polymorphisms of microRNA gene MIR146A were reported as associated with different autoimmune diseases, include systemic lupus erythematosus, psoriatic arthritis, asthma and ankylosing spondylitis. In this study we investigated MIR146A SNPs in Chinese people with ankylosing spondylitis. Three common SNPs: rs2910164, rs2431697 and rs57095329 were selected and genotyped in 611 patients and 617 controls. We found no association between these SNPs and ankylosing spondylitis in our samples.

## Introduction

MicroRNA gene MIR146A plays a significant role in immune system. MIR146A regulates gene expression of TRAF6 and IRAK1 in inflammatory pathway and participates in a negative feedback loop.[1] Case-control studies revealed that MIR146A gene SNPs increased susceptibility in the onset of several autoimmune diseases. Positive results were obtained in systemic lupus erythematosus (SLE), psoriatic arthritis (PsA), asthma and telangiectasia in systemic sclerosis.[2–6] And a recent report mentioned the association between MIR146A SNP rs2910164 and ankylosing spondylitis (AS) in Chinese subjects by Xu et al.[7] In another hand, there were also conflicting negative association reports in PsA, RA and SLE.[8–10] The contribution of MIR146A SNPs to autoimmune diseases needs further investigating.

In this study, we tested frequencies of three common MIR146A SNPs: rs2910164, rs2431697 and rs57095329 in Chinese ankylosing spondylitis cases and controls.

## Subjects and Methods

### Ankylosing spondylitis patients and controls

We collected in total 611 AS patients and 617 healthy controls. All the subjects were unrelated Chinese. The patients were diagnosed by two experienced rheumatologists according to the 1984 New York Modified Criteria. The controls were older than 40 and had no arthritis history. The proportions of male samples in the cases and controls were 78.7% and 77.7%, respectively. The prevalence of HLA-B27 positive was 91.5% in patients and 6.3% in controls.

Written informed consent was received from all participants. This study was approved by the Ethics Review Committee of the Chinese National Human Genome Center at Shanghai. The approval number is 2014–07.

### Genotyping of MIR146A SNPs

Special primer pairs for MIR146A SNPs: rs2910164, rs2431697 and rs57095329 were designed using software Primer3. DNA fragments in all subjects were amplified by PCR. Products were purified with Exon I (New England Biolabs)-SAP (BioTec, Norway), and sequenced on an ABI 3730XL DNA analyzer (Applied Biosystems).

### Statistical Analysis

SNP frequencies were obtained by direct counting. Comparisons of SNP were performed using the Pearson χ^2^ test. Differences in genotype and allele frequencies were calculated using SPSS software.

## Results

We found no significant difference between case and control groups of these three SNPs. Minor allele frequencies in cases and controls were 17.5% vs. 17.9% for rs2431697 C, 19.7% vs. 19.2% for rs57095329 G, and 41.2% vs. 41.1% for rs2910164 G, respectively. The lowest p = 0.439 was observed in rs57095329 for allele test. And allele frequencies of rs2910164 G in case and control groups were nearly equal. For genotypic tests, SNP rs2910164 GG homozygote frequencies in cases and controls were 17.8% and 15.2%, respectively. Chi-square p value for rs2910164 genotype GG vs. GC+CC was 0.235 (Odds Ratio = 1.20, 95% Confidence Intervals 0.89–1.63). Genotype frequencies of all SNPs were in Hardy-Weinberg equilibrium. The statistical power was 0.19 for rs2431697, 0.15 for rs57095329 and 0.17 for rs2910164. See details in Table 1.

**Table 1:** MIR146A gene SNPs distribution in AS and control subjects.

	Allele	AS	Controls	Allele	Genotype	AS	Controls	Genotype p value	
				p value				AA/(Aa+aa)	(AA+Aa)/aa
**rs2431697**	T	1005(82.5)	1012(82.1)	0.806	TT	413(67.8)	420(68.2)	0.888	0.281
n (%)	C	213 (17.5)	220(17.9)		TC	179(29.4)	172(27.9)		
					CC	17(2.8)	24(3.9)		
**rs57095329**	A	976(80.3)	991(80.8)	0.439	AA	391(64.3)	404(65.9)	0.560	0.680
n (%)	G	240(19.7)	225(19.2)		AG	194(31.9)	183(29.9)		
					GG	23(3.9)	26(4.2)		
**rs2910164**	C	708(58.8)	718(58.9)	1	CC	213(35.4)	201(33.0)	0.371	0.235
n (%)	G	496(41.2)	502(41.1)		GC	282(46.8)	316(51.8)		
					GG	107(17.8)	93(15.2)		

Negative results were obtained also in layer analysis by gender. The allele frequencies of rs2910164 G were 41.0% in male patients and 41.2% in male controls (chi-square p = 0.94); 42.7% in female patients and 41.9% in female controls (chi-square p = 0.85). The frequencies of rs2431697 C were 16.0% in male patients and 18.0% in male controls (chi-square p = 0.25); 20.6% in female patients and 19.1% in female controls (chi-square p = 0.69). And the frequencies of rs57095329 G were 19.0% in male patients and 19.8% in male controls (chi-square p = 0.65); 17.8% in female patients and 16.8% in female controls (chi-square p = 0.76).

We compared rs2910164 G frequency between Xu’s and our data. We found no significant difference between two control groups (chi-square p = 0.11). But the allele frequency of rs2910164 G in Xu’s case group (49%, 100 from 2n = 204) was significantly higher than in ours (41%, chi-square p = 0.036). The distributions of this SNP in two case groups were different and caused the dissimilarity between the results.

Common MIR146A gene SNPs were not associated with AS in Chinese in this study.

## Discussion

This time we genotyped three MIR146A common polymorphisms in more than 1200 Chinese people. These SNPs were reported influencing MIR146A gene expression.[2, 11, 12] Although rs2910164 was reported as positive with AS by Xu et al[7], it showed negative in this study. The difference may be caused by sampling from different cohorts. The allele frequencies of rs2910164 in our data were nearly equal in case and control groups (41.1% for G allele) and similar with another reported data from Chinese population (41.0% for G allele from 483 control individuals).[13] The contribution of rs2910164 G to ankylosing spondylitis should be further investigated in more samples.

Up regulated expression of MIR146A were found in different autoimmune diseases, including rheumatoid arthritis (RA), psoriasis, Sjögren's syndrome, and lupus nephritis.[14–17] SNPs rs2910164 C allele reduced the amount of pre- and mature miR146A 1.9- and 1.8-fold, respectively.[12] Frequency of this Pre-RNA allele in Chinese (58.9% in this study) is much higher than that in Europeans (for example 27.1% by Singh et al.).[18] The contribution to autoimmune diseases of this functional microRNA gene SNP may be somehow different between populations. Although MIR146A SNPs did not show relationship with ankylosing spondylitis in this study, it may still involve in the progress of autoimmune diseases development and treatment.

## Supporting Information

S1 File MIR146A genotyping.xlsx. This file includes MIR146A SNPs genotyping information of all AS case and control samples.
(XLSX)
